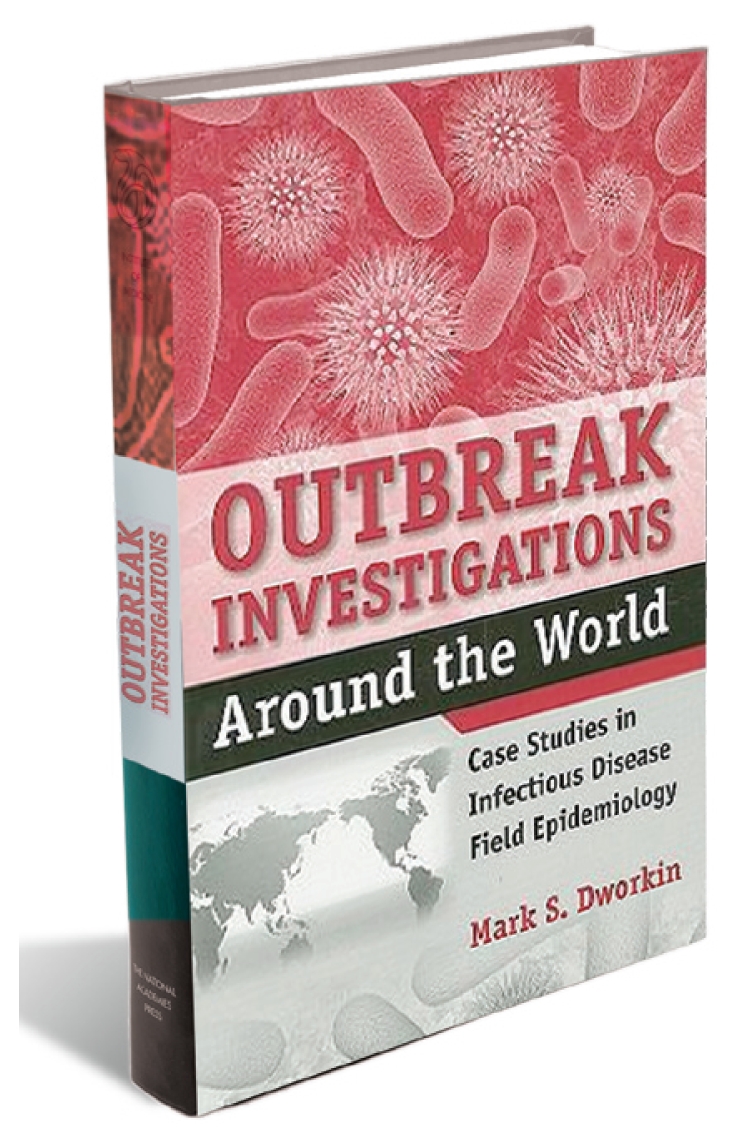# Outbreak Investigations Around the World: Case Studies in Infectious Disease Field Epidemiology

**Published:** 2010-03

**Authors:** Mark L. Wilson

**Affiliations:** *Mark L. Wilson is professor of epidemiology and of ecology and evolutionary biology at the University of Michigan, where he studies environmental and social determinants of infectious disease epidemiology.*

Most people experience infectious disease outbreaks as frightening news reports that cause panic and fear, but for public health workers they are common occurrences that are part of the almost daily routine. Some outbreaks have gained particular notoriety because they were totally unexpected, affected many people, involved a novel microbe, were especially virulent, or for other reasons became a focus of media attention. Although every outbreak has its peculiarities, each represents an important opportunity to understand, generalize, and improve preparedness. *Outbreak Investigations Around the World* is a collection of diverse, mostly well-known case studies presenting both fundamental information and the personal experiences of each chapter’s scientist-author. It represents a compilation of fascinating accounts and insightful lessons for students, professionals, and the public.

Following the editor’s introductory chapter summarizing how outbreaks are investigated, 19 case studies involving actual infectious disease outbreaks from 1964 to 2006 are described. All are germane to problems today. Chapters vary in length and detail, but each presents basic facts about the malady and microbe, as well as the logic, logistics, and luck underlying the investigation. Although the title suggests numerous international settings, most reports are from the United States, with others from Egypt, Gabon, Israel, Liberia, and Portugal. Many chapters contain tables, figures, and photos that transmit the facts and flavor of these initially mysterious events. The 19 diseases involve as many associated pathogens, including viruses (for AIDS, Ebola fever, hepatitis A and B, measles, mumps, whooping cough, yellow fever) and bacteria (anthrax, botulism, legionellosis, leptospirosis, shigellosis, syphilis, toxic shock syndrome). Other diseases caused by protozoa (amebiasis, cholera, cryptosporidiosis) and even a tapeworm (taeniasis) complete the mix. Unlike manuals that present the “facts” of disease symptoms, transmission modes, vaccine availability, or antimicrobial treatment, these vignettes offer some of the same information, but in a style that is easily accessible to a wide audience. The circumstances of outbreak are diverse, including foodborne, waterborne, vectorborne, and airborne transmission, hospital- and restaurant-associated settings, and assorted behavioral, cultural, and environmental contexts. One chapter even describes how a presumed outbreak was misconstrued, and found not to be an outbreak. The variety of different diseases and circumstances is both entertaining and enlightening.

This is not a textbook, but rather a mixture of true stories and instructive histories that students and others will find interesting and informative. Different in depth and detail, the chapters are nevertheless similar for their tenor of uncertainty, surprise, and mystery. Through a personal, storytelling style, each conveys the challenges of the unknown, the urgency of understanding, and the satisfaction of solutions. Chapters from this book could nicely complement more traditional material for courses in infectious disease epidemiology, microbiology, emergency preparedness, or history of medicine. Any teaching effort that addresses the field application of epidemiologic methods could use these investigations to illustrate the many components of research and reporting. The book is equally appealing as supplementary training for public health workers or as easily understood summaries for the curious layperson.

Virtually all of the authors are trained as physicians and worked with the CDC Epidemic Intelligence Service, perhaps suggesting that only health workers with such backgrounds can lead or participate in outbreak investigations. Readers should remember that many important contributions to such efforts are being made by skilled public heath scientists with other preparation and experience, and students should understand that they can pursue various avenues of training and action that are directly relevant to infectious disease epidemiology and prevention. Nearly all chapters convey an appreciation of the difficulties of rapidly obtaining needed information and effectively interpreting it. The importance of working with citizens, community organizations, businesses, nongovernmental organizations, and government agencies is presented as an often critical element of such investigations. Most chapters end with useful “take-home” messages in the form of conclusions or lessons learned. These features all contribute enormously to the educational value of the book, making it much more than a standard text. A major strength of this volume is its portrayal of the excitement and challenge of pursuing the unknown, and ultimately helping to understand and respond to infectious disease threats in the future.

## Figures and Tables

**Figure:**